# DnaJ homolog subfamily A member1 (DnaJ1) is a newly discovered anti-apoptotic protein regulated by azadirachtin in Sf9 cells

**DOI:** 10.1186/s12864-018-4801-z

**Published:** 2018-05-29

**Authors:** Benshui Shu, Jianwen Jia, Jingjing Zhang, Veeran Sethuraman, Xin Yi, Guohua Zhong

**Affiliations:** 1 Key Laboratory of Crop Integrated Pest Management in South China, Ministry of Agriculture, Key Laboratory of Natural Pesticide and Chemical Biology, Ministry of Education, South China Agricultural University, Guangzhou, China; 2 Guangzhou City Key Laboratory of Subtropical Fruit Trees Outbreak Control, Zhongkai University of Agriculture and Engineering, Guangzhou, China; 3 Laboratory of Insect Toxicology, South China Agricultural University, Guangzhou, 510642 China

**Keywords:** Azadirachtin, Apoptosis, 2-DE, Sf-DnaJ1, RNAi

## Abstract

**Background:**

Azadirachtin, one of the most promising botanical insecticides, has been widely used for pest control. Azadirachtin induces apoptosis in insect cell lines, including Sf9, SL-1 and BTI-Tn-5B1–4. Mitochondrial and lysosomal pathways are likely involved in the azadirachtin-induced apoptosis, however, detailed molecular mechanisms remain largely undefined.

**Results:**

Azadirachtin-induced apoptosis in Sf9 cells was verified by morphological observation, Hoechst 33258 staining, and a Caspase-3-based analysis. Comparative two-dimensional gel electrophoresis (2-DE) coupled with a linear ion trap quadrupole (LTQ)-MS/MS analysis identified 12 prominent, differentially expressed proteins following azadirachtin treatment. These differentially expressed genes are involved in regulating cytoskeleton development, signal transduction, gene transcription, and cellular metabolism. Knockdown gene expression of a gene encoding a DnaJ homolog enhanced apoptosis induced by azadirachtin in Sf9 cells.

**Conclusion:**

Azadirachtin treatment induces apoptosis in Sf9 cells and affects expression of multiple genes with functions in cytoskeleton development, signal transduction, gene regulation, and cellular metabolisms. Azadirachtin induces apoptosis at least partially by down-regulation of Sf-DnaJ in Sf9 cells.

**Electronic supplementary material:**

The online version of this article (10.1186/s12864-018-4801-z) contains supplementary material, which is available to authorized users.

## Background

Azadirachtin, a prototypical botanical tetranortriterpenoid isolated from neem trees (*Azadirachta indica*, A. Juss), is one of the most potent botanical chemicals and has been used extensively in pest control [1–3]. Azadirachtin is effective against more than 550 species of insect pests, including insects from Lepidoptera, Hemiptera, Diptera and Orthoptera. The mode of action of azadirachtin against insects include antifeedant effect and disruption of insect growth and development. On the other hand, azadirachtin has little toxicity to mammals and decays fast in the environment [4–6], which makes it a preferred choice for pest management in the field. Azadirachtin is also toxic to cultured insect cells. Inhibition of cell proliferation has been observed in Sf9 cells derived from the ovaries of *Spodoptera frugiperda*, SL-1 cells derived from *Spodoptera litura*, BTI-Tn-5B1–4 cells derived from *Trichoplusia ni*, and C6/36 cells derived from *Aedes albopictus* [7–11]. Treatments of these cells with 10 to 100 nM azadirachtin result in completely inhibition of cell proliferation [7, 8]. Studies with some of the insect cell lines suggest that apoptosis is the cause of cell death based on observed morphological, physiological, biochemical, and toxicological changes [9–12].

The high efficacy of azadirachtin against cultured cells and insects has attracted a great deal of attention to reveal the molecular pathways for its mode of action. However, so far most information on molecular mechanisms associated with azadirachtin toxicity has been obtained from cancer cell lines. Apoptotic signaling pathways are activated in cancer cells following azadirachtin treatments, including the caspase-dependent pathway, AIF-mediated pathway, p38 and JNK1/2 pathway, ROS-dependent MAPK pathway and death receptor pathway [13–15]. In insect cells, the p53 gene is induced in azadirachtin-treated SL-1 cells, resulting in cell cycle arrest and the induction of apoptosis [16]. Using insect Sf9 cells, our group has previously demonstrated that both mitochondrial and lysosomal pathways are involved in apoptosis after azadirachtin treatments [17, 18]. Specifically, we found that cathepsin L released from lysosome to cytosol was induced in azadirachtin-treated Sf9 cells, resulting in the activation of caspase-3 [18]. Despite significant progress has been made, our knowledge on molecular components and pathways leading to apoptosis in azadirachtin-treated cells remains fragmented.

Comparative proteomic analyses are powerful and effective tools for large-scale identification of proteins involved in a specific biological process. Two-dimensional gel electrophoresis (2-DE) combined with mass spectrometry (MS) has commonly used for proteomics and has been extensively applied to analyze the differentially expressed proteins in identical biological samples that are treated differently [19, 20]. For example, 10 proteins of *S. litura* (Fabricius) affected by azadirachtin significantly have been identified using 2-DE, and six of them are functionally assigned based on matrix-assisted laser desorption/ionization time-of-flight (MALDI-TOF-MS) [21]. Two induced hemolymph proteins with functions in lipid metabolism have also been identified using 2-DE coupled with MS/MS from azadirachtin-treated *Ostrinia furnacalis* (Lepidoptera: Crambidae) [22]. Twenty-one differentially expressed proteins have been identified using the 2-DE/MS/MS method in azadirachtin-treated *Drosophila melanogaster* larvae, with results indicating that heat shock protein 23 is the potential target of azadirachtin action [23].

The objective of this study is to conduct a systematic study on proteins expressed differentially in Sf9 cells after treatments with azadirachtin via comparative proteomic analyses. Twelve most differentially expressed proteins were identified by linear ion trap quadrupole (LTQ)-MS/MS. Among these 12 proteins, Sf-DnaJ1 (DnaJ homolog to subfamily A member 1) was down-regulated significantly in azadirachtin-treated Sf9 cells. Knockdown of Sf-DnaJ1 via RNA interference (RNAi) resulted in increased apoptosis in Sf9 cells after azadirachtin treatments. Our results suggest that Sf-DnaJ1 is a regulator of azadirachtin-induced apoptosis.

## Methods

### Chemicals

Hyclone SFX-insect cell culture medium was purchased from Thermo Scientific (USA), and fetal bovine serum (FBS) was purchased from Gibco (USA). IPG drystrip and IPG buffer were purchased from Amersham Biosciences (Uppsala, Sweden). Azadirachtin (95%) was obtained from Sigma (St.Louis, MO, USA). Other chemicals were domestic products with analytical grade. Rabbit polyclonal antibodies against HSP40, TCTP and GAPDH, respectively, were obtained from BOSTER (Wuhan, China).

### Cell culture

Sf9 cells were obtained from the State Key Laboratory for Biocontrol/Institute of Entomology, Sun Yat-Sen University (Guangzhou, China), and were maintained at 27 °C in 25 cm^2^ culture flasks (Corning, USA) containing 3 mL Hyclone SFX-insect cell culture medium supplemented with 5% fetal bovine serum. The doubling time under optimum conditions was 18–24 h and cells were subcultured every 2 days.

### Cell viability assay

Sf9 cells were seeded onto a 96-well plate (5 × 10^3^/well) and incubated for 24 h, then exposed to a series of concentrations of azadirachtin for 24 h and 0.1% DMSO was included as a control. Fifty μL of methylthiazoletetrazolium (MTT) solution was added to each well and cells were incubated in dark for another 4 h. After removing the supernatant, 150 μL DMSO was added and mixed thoroughly with the pipette. Cell viability was measured based on absorbance at 490 nm using a microplate reader (Thermo Scientific, Waltham, MA, USA).

### Morphological observation by inverted phase contrast microscopy

Cells seeded in 6-well plates were treated with 0.75 μg/mL azadirachtin and 0.1% DMSO was used as control. Morphological characteristics of cells at 0, 24, 48, and 72 h after treatments were recorded by an inverted phase contrast microscope (Lecia, Japan), respectively.

### Hoechst 33258 analysis

Hoechst 33258 is a blue fluorescent dye that could penetrate cell membranes and stain the cell nuclei with blue color. Sf9 cells treated with azadirachtin for 24, 36 and 48 h were stained with 0.5 mL Hoechst 33258 solution for 5 min and then washed with phosphate-buffered saline (PBS) twice for 3 min each time. The stained cells were observed under the fluorescent microscope (Nikon, Japan).

### Analysis of caspase-3-like enzymatic activity

Sf9 cells treated with azadirachtin were collected and the caspase-3-like proteolytic activity was measured using a Caspase-3 Colorimetric Assay Kit (KeyGEN BioTECH, Nanjing, China). The cells were then washed with PBS twice and collected by centrifugation at 2000 rpm for 5 min. Total cellular proteins were extracted using a cold lysis buffer on ice for 20–60 min. Protein concentrations were determined following the Bradford approach [24]. The solution containing 150 mg proteins with the Caspase-3 substrate (integrating specific luminescence substrate) was incubated in dark at 37 °C for 4 h. Caspase-3-like enzymatic activity was measured based on the absorbance of samples measured at 405 nm using a microplate reader (Thermo Scientific, USA). The Cacpase-3 inhibitor Z-VAD-FMK was also used with the final concentration of 20 μM.

### Preparation of protein samples

The adherent Sf9 cells treated with 0.75 μg/mL azadirachtin for 24 h and control group were washed with PBS twice and then mixed with 1 mL lysis buffer containing 40 mM Tris-base, 7 M urea, 2 M thiourea, 4% (*w*/*v*) CHAPS, 2% (*v*/v) carrier ampholytes pH 3–10 and 65 mM DTT. The homogenates were shaken for 15 min in an ice-water bath and centrifuged at 14000 rpm for 15 min at 4 °C. Protein concentrations of supernatants were determined by the Bradford method.

### 2-DE, gel staining and image analysis

Before loading for 2-DE, samples were dissolved in 350 μL rehydration buffer containing 7 M urea, 2 M thiourea, 4% (w/v) CHAPS, 2% (v/v) IPG buffer, 20 mM DTT, and a trace of bromophenol blue, then centrifuged at 14000 rpm for 5 min. Total protein extracts from control and treated samples were separated through 2-DE. Two protein samples (140 μg) were loaded onto analytical and preparative gels, respectively. Isoelectric focusing (IEF) was carried out on an IPGphor system (Amersham Biosciences) with pH 4–7 IPG strips (18 cm, linear) according to the manufacturer’s instructions. A total of 60 kVh was applied. Then the IPG strips were equilibrated in 3 mL equilibration buffer twice for 15 min. The first equilibration was performed in a buffer containing 50 mM Tris-HCl (pH 8.8), 6 M urea, 30% (*v*/v) glycerol, 2% (*w*/*v*) SDS, 1% (w/v) DTT, and a trace amount of bromophenol blue. The second equilibration was performed in a buffer modified by 2.5% w/v IAA instead of DTT. The strips were placed on the top of 12.5% SDS-polyacrylamide gels and sealed with 0.5% agarose. Electrophoresis was carried out on a Hoefer SE 600 apparatus (Amersham Biosciences) at 20 °C with the current of 15 mA/gel for 40 min, and then 45 mA/gel for 6 h. The protein spots in gels were visualized by staining with silver nitrate [25]. At least three replicates were performed for each sample. Images of each gel were acquired using Lab-Scan version 3.0 software (GEHealthcare) on an Image-Scanner. Images were analyzed by ImageMaster 2-DE platinum version 5.0 software. The intensity of the protein spots was calculated with PDQuest 8.0 software.

### Protein digestion, LTQ-MS/MS and database searching

In order to locate protein spots with different intensity in gels, Coomassie Brilliant Blue G-250 was used to stain the gels for mass spectrometric analysis. The gels were fixed with a buffer containing 40% ethanol and 10% glacial acetic acid for 1 h, washed with double distilled water three times, stained with Coomassie Brilliant Blue G-250 staining solution overnight, and decolorized in a destaining solution for at least 4 h. The significantly altered protein spots were located by comparing gels with control and treated samples side by side. The identified protein spots were cut out from the gel, further destained with 30 mM potassium ferricyanide/100 mM sodium thiosulfate (1:1, *v*/v) for 20 min, and washed in Milli-Q water until the gels were completely destained. The spots were kept in 0.2 M NH_4_HCO_3_ for 20 min and then lyophilized. Each spot was digested in 12.5 ng/mL trypsin with 0.1 M NH_4_HCO_3_ overnight. The peptides were extracted with 50% Acetonitrile, and 0.1% TFA three times.

Separation and identification of the digested proteins were conducted on a Finnigan LTQ mass spectrometer (ThermoQuest, San Jose, CA, USA) coupled with a Surveyor HPLC system (ThermoQuest, San Jose, CA, USA). A Microcore RP column (C18 0.15 mm × 120 mm; ThermoHypersil, San Jose, CA, USA) was used to separate the protein digests. Solvent A was 0.1% (*v*/v) formic acid, and solvent B was 0.1% (v/v) formic acid in 100% (v/v) ACN. The gradient was held at 2% solvent B for 15 min, and increased linearly to 98% solvent B for 90 min. The peptides were eluted from the C18 microcapillary column at a flow rate of 150 μL/min and then electrosprayed directly into an LCQ-Deca mass spectrometer with the application of spray voltage of 3.2 kV and capillary temperature at 200 °C. The full scan was ranged from M/Z 400 to 2000. Protein identification based on MS/MS data was performed with SEQUEST software (University of Washington, licensed to Thermo Finnigan) based on the database of Swiss Port. The species for sequence search is Lepidoptera. Protein identification results were filtered with a stringent filter condition of Xcorr (1 + ≥ 1.9, 2 + ≥ 2.2, 3 + ≥ 3.75) and DelCn (≥ 0.1).

### Identification and sequencing of sf-DnaJ1 cDNA

To obtain a full length Sf-DnaJ1 cDNA, total RNA was isolated from Sf9 cells with an E.Z.N.A.™ Total RNA Kit II (OMEGA, USA) according to the manufacturer’s instructions. First strand cDNAs were synthesized using a PrimeScript® 1st Strand cDNA Synthesis Kit (TaKaRa) according to the provided protocol. cDNA of Sf-DnaJ1 was amplified by PCR with the degenerate primers Sf-DnaJ1-F and Sf-DnaJ1-R (Additional file 1: Table S1) and 50 μL reaction mixture contained 0.5 μL template, 1 μL of each 10 mM primer, 0.5 μL Taq DNA polymerase (TIANGEN, Beijing, China), 4 μL of 2.5 mM dNTP mixture (TIANGEN, Beijing, China), and 5 μL 10× Taq Buffer. The PCR program was performed with 32 cycles of 30 s at 94 °C, 30 s at 55 °C and 30 s at 72 °C. The 5’-RACE (SMART RACE, Clontech) and 3’-RACE (TaKaRa) methods were used to fulfill the full-length cDNA of Sf-DnaJ1. To ensure the 5′ and 3′ fragments were cloned from the same gene, specific primers were designed and PCR was used to amplify the coding region of the transcript encoding Sf-DnaJ1.

### Quantitative real time PCR

In order to confirm the expression profiles of six identified proteins, quantitative real-time PCR (qRT-PCR) was performed. Total RNA was extracted from Sf9 cells treated with azadirachtin for 24 h and control cells using a Total RNA Kit II (OMEGA, USA). The cDNA for qRT-PCR was synthesized using a PrimeScript™ RT reagent Kit (TaKaRa, Japan), which has a gDNA Eraser to eliminate DNA contamination. qRT-PCR was performed on CFX Connect™ Real-Time System (Bio-Rad, USA) using SsoAdvanced™ SYBR® Green Supermix (Bio-Rad, USA). The PCR was carried out as follows: 95 °C for 3 min for denaturation, 40 cycles of 95 °C for 10 s, 60 °C for 10 s, 72 °C for 30 s, and a dissociation step at the end. GAPDH was used as a reference for normalization. Relative expression levels were calculated by the 2^−ΔΔCT^ method. Primers used in the experiments are listed in Additional file 1: Table S1.

### Western blot assays

Cells were collected and washed with PBS. Total cellular proteins were extracted using the CytoBuster™ Protein Extraction Reagent (Novagen, USA) according to the manufacturer’s protocol. Protein concentrations were determined by the Bradford method. Equal amounts of proteins from different samples were separated on a 12% SDS-PAGE gel. Proteins in the gel were then transferred to a polyvinylidene difluoride membrane (PVDF, Millipore, USA). The membrane was washed with TBS for 3 times, incubated with TBS supplemented with 5% fat-free milk at 4 °C overnight, and incubated with HSP 40 antibody, TCTP antibody or GAPDH antibody at room temperature for 2 h. Subsequently, the membrane was washed and incubated with the peroxidase-conjugated secondary antibody at room temperature for more than 2 h. The protein bands were detected by the enhanced chemiluminescence western blot kit (CW0049, CWBIO, Beijing, China) and detected by exposure to X-ray film a dark room.

### Double-stranded RNA synthesis and transfection

dsRNA against the Sf-DnaJ1 transcript was synthesized using a T7 RiboMAX™ Express RNAi System (Promega, USA) according to the provided protocol. Template DNA was amplified by PCR with the RNAi primers listed in Additional file 1: Table S1. The dsRNA against egfp, which used as the negative control, was similarly synthesized by the template pEGFP-C and primers in Additional file 1: Table S1. The size and integrity of dsRNAs were checked by agarose gel electrophoresis.

Transfection of Sf9 cells was performed based on the Lipofectin transfection method. Monolayer cultures of Sf9 cells were prepared in 35-mm cell culture dishes (Corning, USA). Transfection was carried out by incubation with 2 mL Hyclone SFX-insect cell culture medium (without FBS) containing 5 μg dsRNAs overnight at 27 °C, followed by incubation in 10 μL lipofectamine 2000 (Invitrogen) for 6 h. The medium was then replaced with a medium containing FBS. After 24 h treatment, the RNAi efficiency was examined based on qRT-PCR and western blot results.

### Annexin V-FITC/propidium iodide double-staining and flow cytometry

Anchorage-dependent Sf9 cells treated with 0.75 μg/mL azadirachtin for 24 h were collected by centrifugation at 2000 rpm for 5 min at 4 °C. The cells were then resuspended and washed twice with PBS. The cells were then fixed in 500 μL binding buffer. Prior to cytometry analysis, 5 μL Annexin V-FITC and 5 μL PI were added to the fixed cells which were then incubated in dark for 15 min at room temperature. The cells were analyzed through a flow cytometry with an Ar laser with excitation and emission wavelengths 488 nm and 530 nm, respectively. At least 2.0 × 10^4^ cells were counted in each assay. The Sf9 cells with Sf-DnaJ1 knocked down and GFP control cells were treated with 0.75 μg/mL azadirachtin for 24 h and used for analyses.

### Data analysis

Each treatment had three replicates and data were expressed as the mean values ± SEM. One-way ANOVA followed with Duncan’s new multiple range test (DMRT) and student’s *t* test were conducted during statistical analyses (*P* < 0.05).

## Results

### Azadirachtin inhibited cell viability and proliferation

**Fig. 1:**
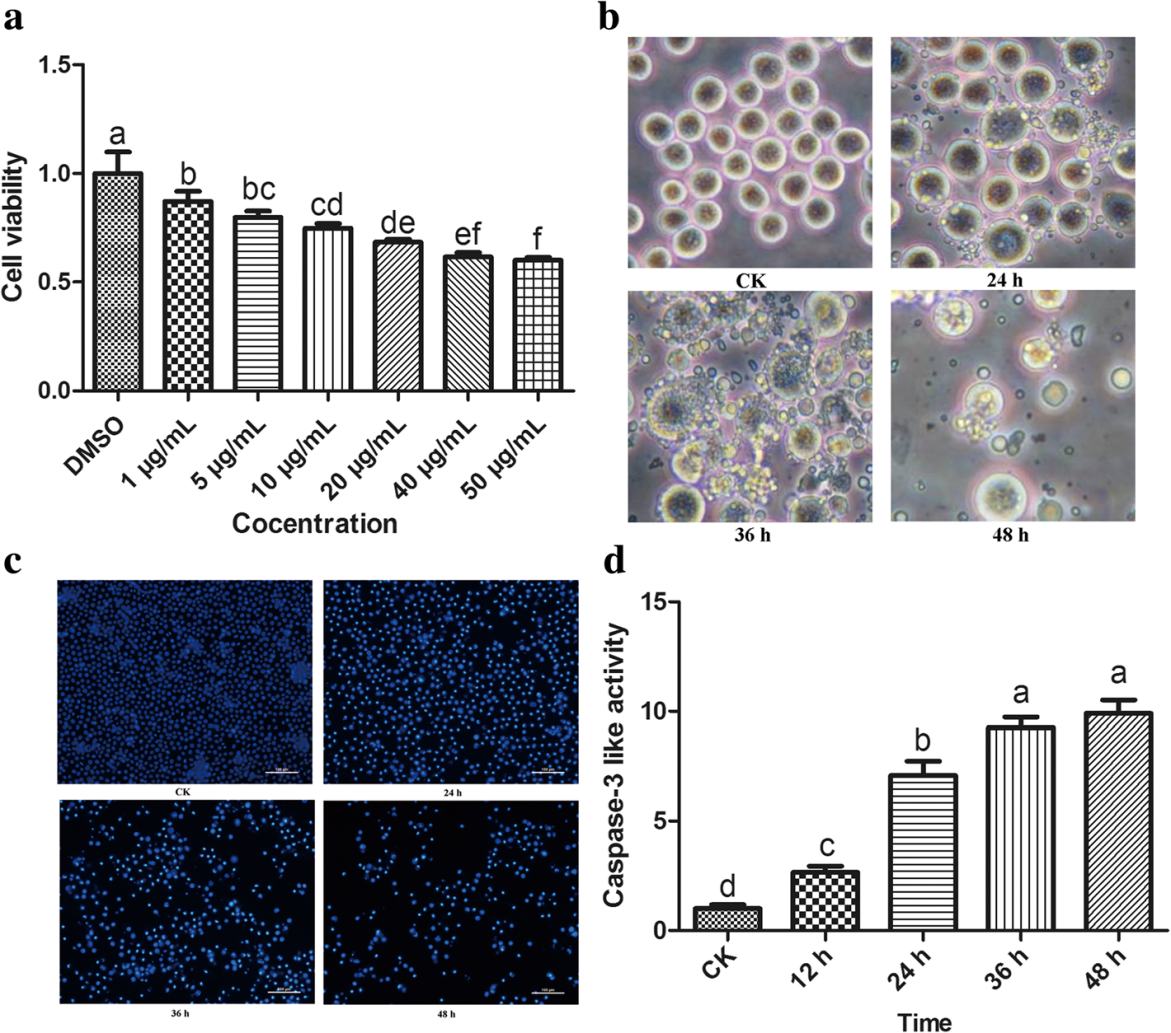
The analysis of proliferation inhibition and apoptosis induction by azadirachtin in Sf9 cells. **a** Cell viability of Sf9 cells after treated with multiple concentrations of azadirachtin for 24 h. **b** Morphological changes in Sf9 cells treated with 0.75 μg/mL azadirachtin at different times. **c** Cell nucleus morphology was detected by Hoechst 33258 staining. **d** The detection of Caspase-3 like activity in Sf9 cells after 0.75 *μ*g/mL azadirachtin treatment for multiple time points. Different letters above bars indicate significant differences between different treatments by ANOVA followed by DMRT test (*P* < 0.05)

As shown in Fig. 1a, the inhibition rates of Sf9 cell proliferation were 14.6 ± 2.21%, 23.1 ± 3.32%, 28.7 ± 2.44%, 36.1 ± 1.56%, 43.8 ± 2.25% and 45.5 ± 1.35%, respectively, after cells treated with azadirachtin at the concentrations of 2, 5, 10, 20, 40, 50 μg/mL for 24 h. These results revealed that azadirachtin had a strong inhibition effect on cells proliferation and decreased cell viability in a dose-dependent manner.

### Morphological changes associated with apoptosis

Our previous study showed that a low concentration of azadirachtin induced apoptosis of Sf9 cells [12]. As shown in Fig. 1b, the morphological changes of Sf9 cells treated with 0.75 μg/mL azadirachtin could be observed clearly under the inverted phase contrast microscopy. Typical morphological characteristics of apoptosis were displayed in Sf9 cells treated with azadirachtin for 24 h, including cell shrinkage, increased gaps, membrane blebbing and apoptotic bodies. After treatment for 48 h, gaps of cells increased and apoptotic bodies occurred widely. The number of viable cells and apoptotic bodies reduced greatly after treatment for 72 h, and few cells kept the normal morphological appearance. These results suggest that typical morphological characteristics of apoptosis are induced in cells treated with 0.75 μg/mL azadirachtin in Sf9 cells.

### Azadirachtin induced nuclear condensation

As shown in Fig. 1c, Hoechst 33258 staining revealed nuclear changes induced by azadirachtin in Sf9 cells. The nuclei of control cells exhibited uniform sizes and were stained homogeneous. In comparison, nuclei of azadirachtin-treated cells exhibited deeper blue staining with nuclear and chromatin condensation after treatment for 24, 36 and 48 h, respectively. The number of live cells decreased gradually and nuclear condensation became more obvious with longer treatment time.

### Azadirachtin increased caspase-3-like proteolytic activity

In order to examine if caspases were activated in Sf9 cells treated with azadirachtin, caspase-3-like proteolytic activity was determined. Compared with that in control cells, the caspase-3-like proteolytic activity increased 2.66, 7.08, 9.26 and 9.91 fold in cells treated with azadirachtin for 12, 24, 36 and 48 h, respectively (Fig. 1d). Apoptosis induced by azadirachtin was inhibited completely by Z-VAD-FMK and the caspase-3-like proteolytic activity in cells treated with both azadirachtin and Z-VAD-FMK was similar to normal cells (Additional file 2: Figure S1). These results indicate that azadirachtin induce apoptosis via activating caspase-3 activity in a time-dependent manner.

### Identification of differential expressed proteins

**Fig. 2:**
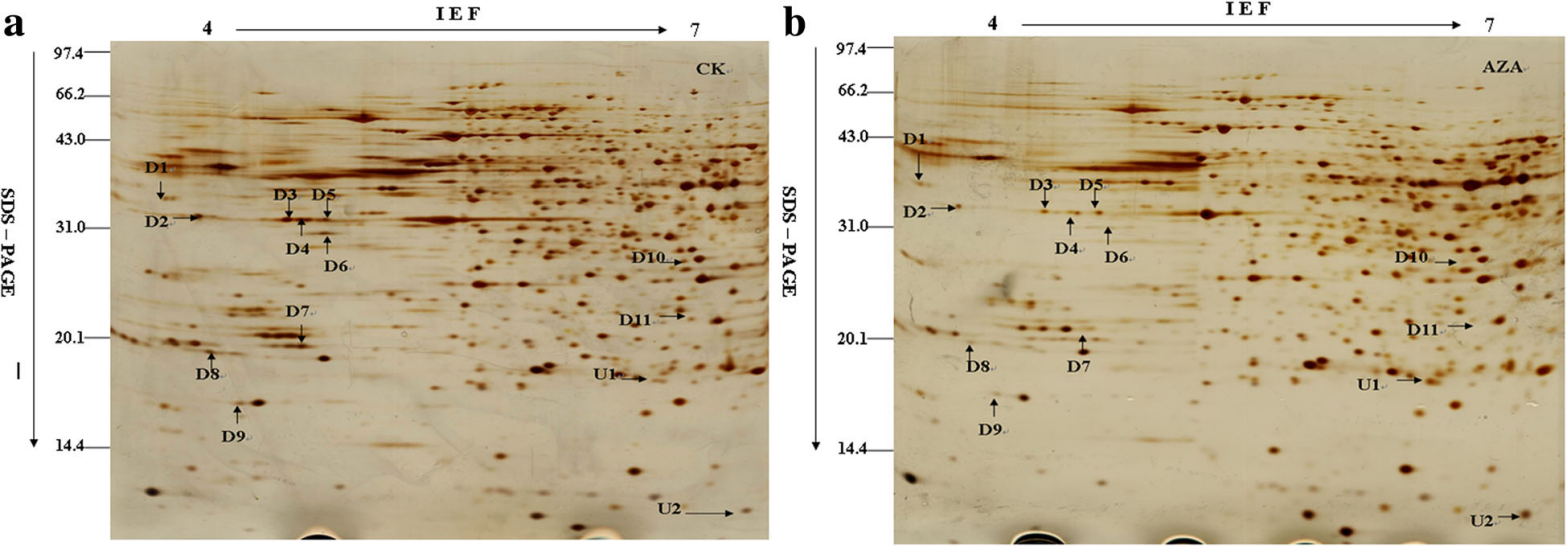
The two-dimensional electrophoresis images of azadirachtin-treated Sf9 cells. **a** 2-DE gel of control sample. **b** 2-DE gel of the sample treated with 0.75 *μ*g/mL azadirachtin for 24 h. Arrows indicate the differentially expressed protein spots, which were designated as D1–D11 and U1, U2. Among them, spots D1-D11 are down-regulated and spots U1and U2 are up-regulated

**Fig. 3:**
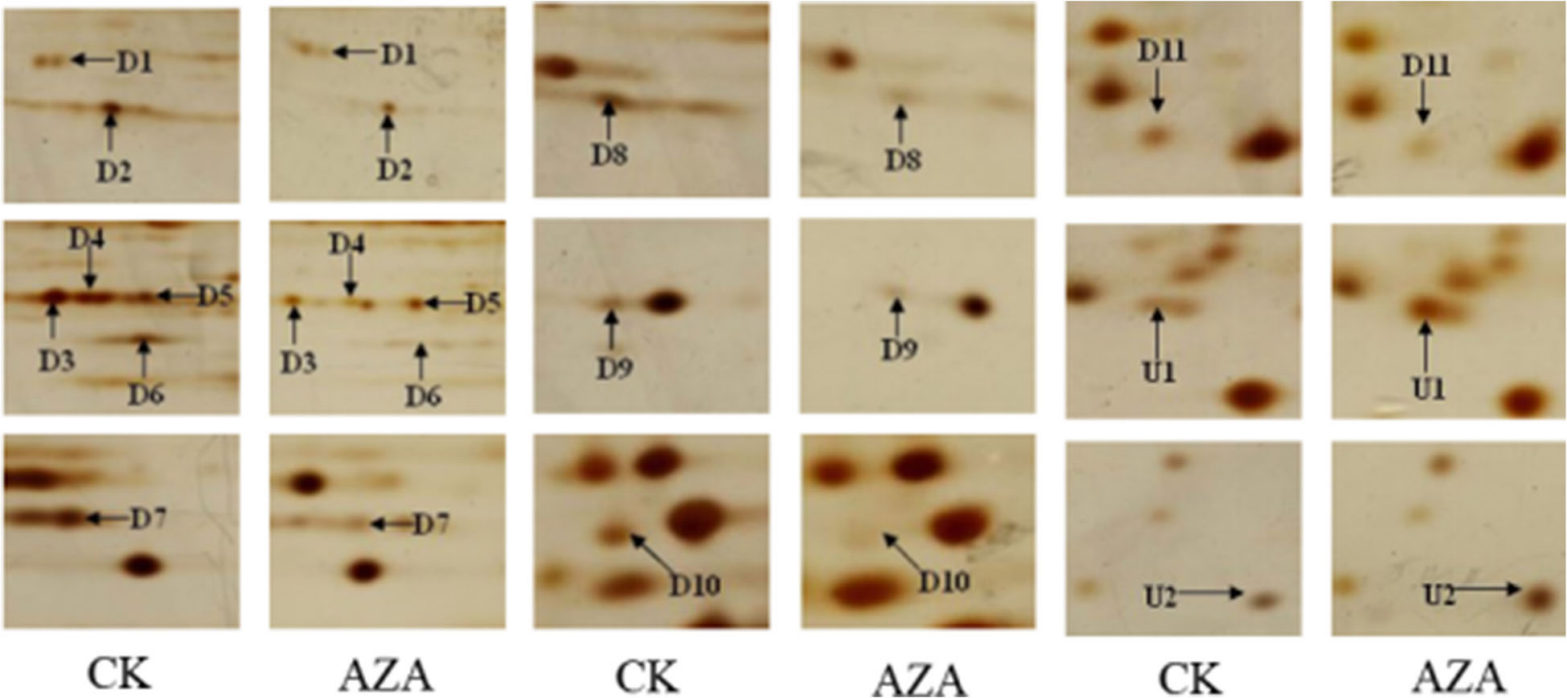
The specific figures of different spots

Comparative proteomic analyses were performed to identify azadirachtin-responsive proteins. Approximately 800 protein spots were detected on a 2-D gel using the Image Master 5.0 software. The criterion for proteins with significant changes in abundance was at least a 1.5-fold increase or decrease between control cells and cells treated with azadirachtin for 24 h. Thirteen protein spots indicated by arrows satisfied this criterion and were considered differentially expressed proteins (Fig. 2). Relative intensity of these proteins was showed in Additional file 3: Figure S2. Abundance of proteins corresponding to spots D1 to D11 decreased in treated cells whilst abundance of proteins corresponding to spots U1 and U2 increased in treated cells (Fig. 3).

The identified azadirachtin-responsive proteins are listed in Table 1 and the matched peptide sequences are listed in Additional file 4: Table S2. Proteins up-regulated by azadirachtin were ribosomal protein L9 (Sf-RpL9, U1) and abnormal wing disc-like protein (Sf-AWD, U2). Proteins down-regulated were DnaJ homolog subfamily A member1 (Sf-DnaJ1, D1), ribosomal protein SA (Sf-RpSA, D2), actin (D3-D5), beta-tubulin (D6), proteasomezeta subunit (Sf-PS, D7), P27BBP/eIF6-like (Sf-P27BBP/eIF6, D8), translationally-controlled tumor protein homolog (Sf-TCTP, D9), Proteasome subunit alpha type 6-A (Sf-PMSA6, D11), and an unknown protein (D10).

**Table 1:** Identification of differentially expressed proteins in Sf9 cells treated with the azadirachtin by LTQ-MS/MS

Spot no	Protein name	Fold change ^a)^	NCBI accession number	DP ^b)^	AAC ^c)^ (%)	MW (Da)	PI	Peptides identified
D1	DnaJ homolog subfamily A member 1	1.61 ± 0.13	gi|14053203	2	2.00	45134.93	6.38	SGNDLILR
D2	Ribosomal protein SA	2.85 ± 0.18	gi|54609281	3	7.84	33408.77	4.87	FAAHTGATPIAGR
D3	Actin	8.97 ± 0.21	gi|46371991	6	13.70	40603.37	5.46	GYSFTTTAER
D4	Actin	3.21 ± 0.11	gi|46371991	7	16.99	40603.37	5.46	GYSFTTTAER
D5	Actin	1.92 ± 0.08	gi|46371991	7	16.99	40603.37	5.46	GYSFTTTAER
D6	Beta-tubulin	10.43 ± 0.24	gi|74275413	3	10.14	33323.12	5.9	EVDEQMLNIQNK
D7	Proteasome zeta subunit	6.26 ± 0.23	gi|114050993	7	36.63	26874.64	4.98	LFQVEYAIEAIK
D8	P27BBP/eIF6-like	3.17 ± 0.14	gi|82880642	4	14.69	26304.74	4.63	VQFENNNEVGVFSK
D9	Translationally-controlled tumor protein homolog	2.38 ± 0.16	gi|74837218	10	41.86	19938.81	4.67	LVETYAFGDKK
D11	Proteasome subunit alpha type 6-A	1.73 ± 0.07	gi|114052160	3	13.41	27143	6.44	GTDAAVVAAQR
U1	Ribosomal protein L9	5.06 ± 0.19	gi|112983495	6	25.79	21377.03	9.94	MAPGVTVVNSPK
U2	Abnormal wing disc-like	2.80 ± 0.22	gi|153791847	5	27.92	17312.92	6.75	NIIHGSDSVESAK

a) Fold change: D1-D11: the ratio of protein intensity of CK versus AZA; U1-U2: the ratio of protein intensity of AZA versus CK.

b) Distinct peptides matched. c) Amino acids coverage.

### Characterization of sf-DnaJ1transcript

A full length cDNA encoding Sf-DnaJ1 was cloned and sequenced as described in Materials and Methods. The nucleotide sequence was deposited in GenBank with the accession number KF562156. The Sf-DnaJ1-encoding cDNA is 1757 bp long with 148 bp at 5′- and 397 bp at the 3′-untranslated regions. The predicted protein has 404 amino acid residues with calculated molecular mass 45.46 kDa. The predicted Sf-DnaJ1 share 89, 77 and 67% sequence identity with DnaJ family proteins from *Bombyx mori*, *Tribolium castaneum* and *Aedes aegypti*, respectively. The phylogenetic relationship of Sf-DnaJ1 with paralogs from other species is shown in Additional file 5: Figure S3.

### Validation of differentially expressed proteins by qRT-PCR and western blot

**Fig. 4:**
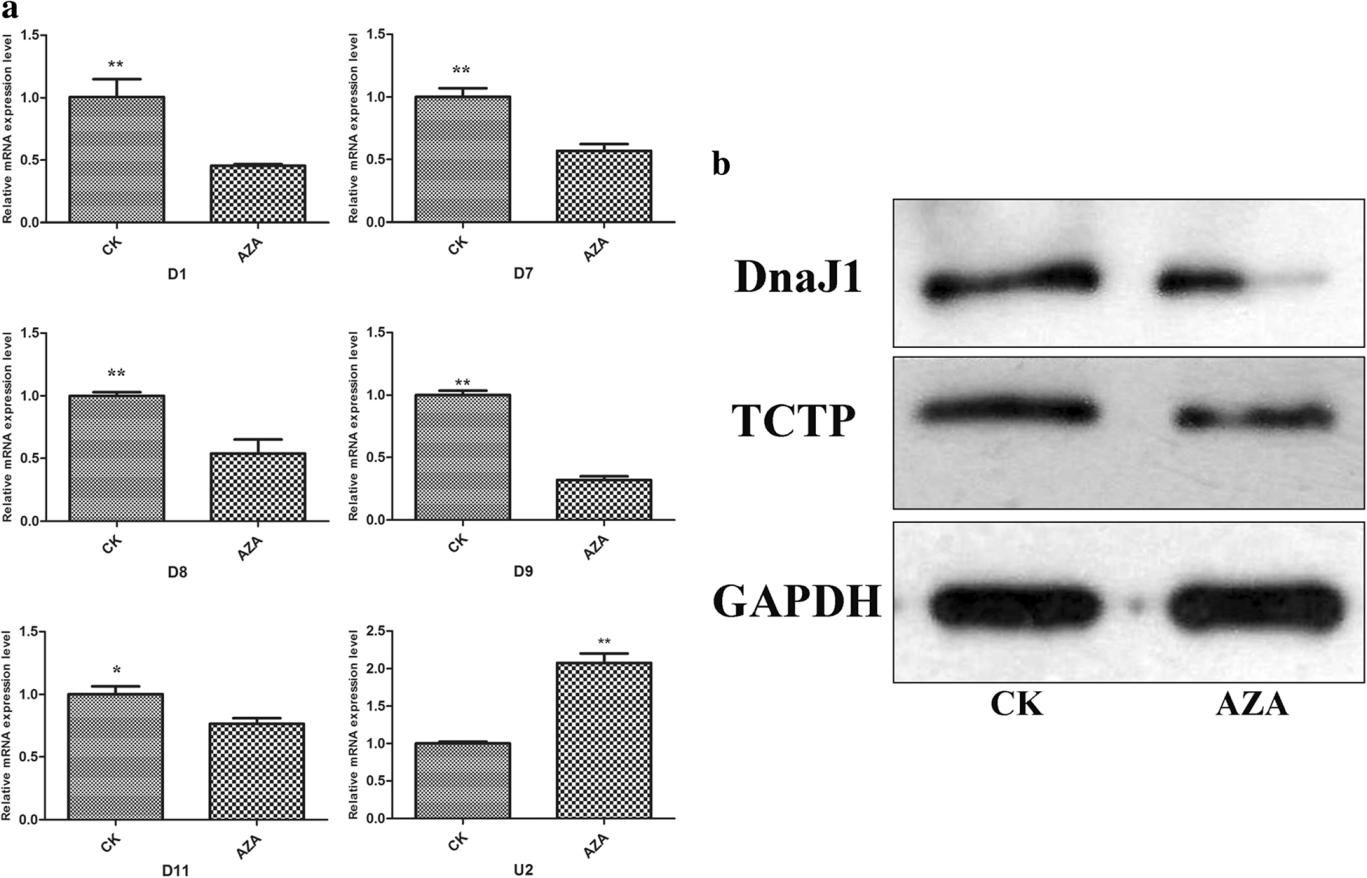
The expression level changes of differentially expressed proteins verified by qRT-PCR and western blot. **a** The mRNA expression profiles of six differentially expressed proteins in Sf9 cells with azadirachtin treatment for 24 h. Cells that treated with 0.1% DMSO were used as control. The GAPDH gene was used as the reference gene and data are expressed as arithmetic mean ± SEM of three independent experiments. Different letters above bars indicate significant differences between different treatments by ANOVA followed by student’s *t* test (P < 0.05). **b** the protein expression changes of DnaJ1 and TCTP in Sf9 cells after azadirachtin treatment for 24 h

To examine the expression of the six genes affected by azadirachtin at transcriptional level, qRT-PCR was conducted. Consistent with the proteomic analysis, the levels of the transcripts encoding Sf-DnaJ1, Sf-PS, Sf-P27BBP/eIF6, Sf-TCTP and Sf-PMSA6 decreased, whereas the level of the transcript encoding Sf-AWD increased in azadirachtin-treated Sf9 cells. Specifically, the level of transcripts encoding Sf-DnaJ1, Sf-PS, Sf-P27BBP/eIF6, Sf-TCTP and Sf-PMSA6 decreased by 39.2, 43, 47, 57.1and 23.7%, respectively (Fig. 4a), while the level of the transcript encoding Sf-AWD increased 107.6%. Western blot analysis revealed that DnaJ1 and TCTP decreased in Sf9 cells treated with azadirachtin for 24 h.

### Knockdown of sf-DnaJ enhanced apoptosis induced by azadirachtin

**Fig. 5:**
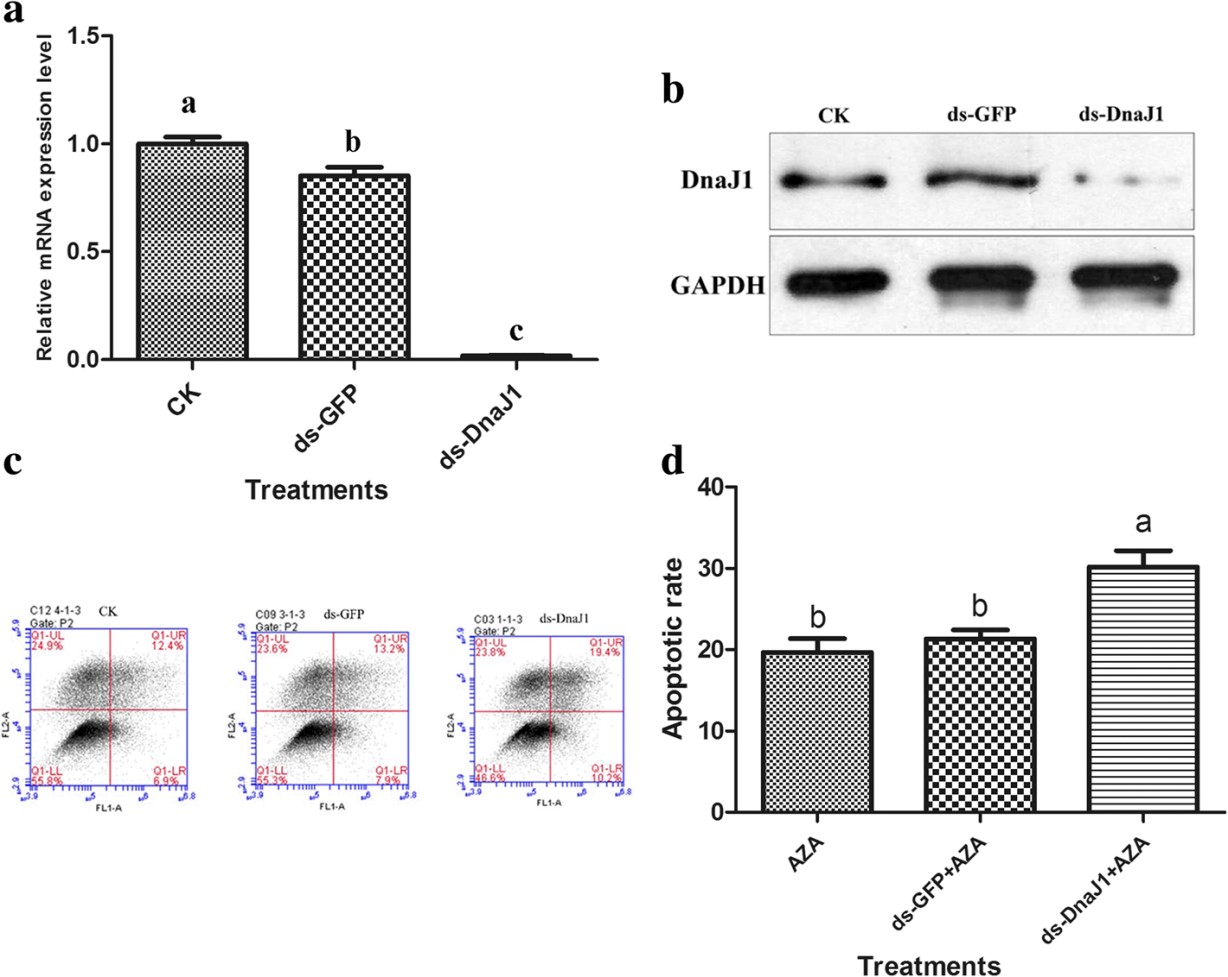
Silence of Sf-DnaJ1 enhanced apoptosis induced by azadirachtin. **a** The relative mRNA expression level changes of Sf-DnaJ1 in Sf9 cells with RNAi treatments. Control, normal cells; negative control, cells treated with unrelated dsRNA. **b** The protein expression levels of DnaJ1 in Sf9 cells with RNAi treatments. **c** The effects of RNAi treatments on apoptosis induced by azadirachtin in Sf9 cells were detected by flow cytometry. **d** The effects of RNAi treatments on apoptotic rate induced by azadirachtin in Sf9 cells. The data represent the mean values± S.E.M of three independent experiments. Different letters above bars indicate significant differences between different treatments at the same time (a represent *p* < 0.05) by ANOVA followed by DMRT

To further confirm the role of Sf-DnaJ1 in azadirachtin-induced apoptosis, dsRNA against Sf-DnaJ1 transcripts was used for gene silencing. The transcript and protein levels of Sf-DnaJ1 were reduced significantly (98.2%) in dsRNA-treated cells compared to controls (Fig. 5a and b). In addition, the cells treated with ds-DnaJ1 could not induced apoptosis and the apoptotic rate had no significant difference with normal cells (Additional file 6: Figure S4). However, silencing the ds-DnaJ1-encoding gene resulted in the enhancing of azadirachtin’s effect on apoptosis (Fig. 5c, d).

## Discussion

Azadirachtin has been proven to be an effective insecticidal ingredient for pest management and has the ability to induce apoptosis in cultured cell lines [26]. In this study, Sf9 cells treated with 0.75 μg/mL azadirachtin exhibited morphological changes typical of apoptosis, as have been previously observed in other insect cells in vitro [11, 17]. Apoptosis is a form of physiological cell death with important roles in various biological processes [27]. Although it has been well documented that apoptosis is associated with azadirachtin treatments, the information on the molecules and pathways affected by azadirachtin remained fragmented. In order to identify key regulators and uncover the molecular mechanism of apoptosis induced by azadirachtin, proteomic analyses including 2-DE coupled with MS-based protein identification was carried out. Twelve proteins were identified with significant changes after azadirachtin treatment (Fig. 2).

One of the down-regulated proteins was identified as DnaJ homolog subfamily A member 1 (alternative name: heat shock 40 kDa protein 4, hsp40–4). Heat shock proteins (HSPs) have been recognized as common molecular chaperones induced under various stress conditions, including heat shock, heavy metals, exposure to radiation, ethanol, oxidative stress and so on [28]. Previous studies have provided substantial evidence that HSPs may regulate apoptosis. Hsp27 and Hsp70 have effects as anti-apoptotic proteins [29, 30]. Hsp60 and Hsp10 can enhance the proteolytic maturation of caspase-3 [31]. Inhibition of Hsp70 and Hsp40 by HSP inhibitor KNK437 results in enhanced PS-341-induced cell death [32]. Hsp40–Hsp70 pair plays a counter role through interacting with Bax, resulting in the inhibition of its translocation to mitochondria in NO-induced apoptosis [33]. HSP 90 has been identified as a potential target of azadirachtin effect through reversing docking [34]. Hsp40 has been characterized as a co-chaperone involved in regulation of Hsp70 chaperone activity, but it remains unclear whether it can regulate apoptosis independently [35, 36]. In this study, we provided two pieces of evidence that, for the first time, demonstrated that a Hsp40 member (Sf-DnaJ1) regulates apoptosis. First, we found that the protein abundance of Sf-DnaJ1 decreased significantly in Sf9 cells treated with azadirachtin, indicating that Sf-DnaJ1 was one of the regulators of azadirachtin action. Second, silencing Sf-DnaJ1-encoding gene enhanced the capability of azadirachtin on inducing apoptosis. Our results indicated that Hsp40 can be an independent apoptosis regulator. Further studies are required to reveal the molecular pathway between azadirachtin and Sf-DnaJ1, and downstream steps.

Previous studies have shown that azadirachtin could induce the depolymerization of actin, leading to cell arrest and subsequently apoptosis in a caspase-independent manner [37, 38]. Our results provide further support to this observation. Compared with untreated control cells, the abundance of actins (three protein spots corresponding to D3, D4 and D5) and β-tubulin (D6) decreased dramatically in Sf9 cells treated with azadirachtin, suggesting that azadirachtin exerted its effects by inhibiting actin and tubulin to remodel apoptotic cells.

Proteasomes are ubiquitous and abundant multi-catalytic enzyme complexes in charge of the degradation of most intracellular proteins [39]. A previous study has showed that a ubiquitin-proteasome complex is involved in the apoptotic process [40]. Proteasomes can stabilize pro-apoptotic proteins including p53 and Bax in various cell types, but they also could suppress apoptosis induced by some stimuli [41–44], suggesting that the roles of proteasomes in apoptosis are complex and can go to either direction. In our results, the abundance of proteasome subunits zeta (D7) and alpha type 6-A (D11) decreased in Sf9 cells after azadirachtin treatment, indicating that azadirachtin could be a new proteasome inhibitor.

Translationally-controlled tumor protein homolog (TCTP) is generally regarded as a highly conserved protein with multiple functions. It is widely expressed in various tissues or cell types and plays an important role in maintaining survival of a variety of cell types [45]. Several lines of evidence indicate that TCTP has a role as an anti-apoptotic protein in cultured cells and it protects cells from apoptotic cell death by inserting into the mitochondrial membrane and inhibiting the dimerization of Bax [46]. TCTP probably interacts selectively with actin and microtubule cytoskeleton to regulate cell shape during interphase and mitosis [47]. TCTP inhibits apoptosis by controlling the stability of tumor suppressor p53, which in turns represses the transcription of the TCTP-encoding gene [48, 49]. In this study, the abundance of TCTP (D9) decreased in Sf9 cells treated with azadirachtin, indicating that azadirachtin could regulate the proliferation and apoptosis of Sf9 cells via inhibiting the gene expression of TCTP.

The abundance of three ribosome proteins was affected by azadirachtin. Azadirachtin reduced the abundance of ribosomal protein SA (D2) and P27BBP/eIF6-like (D8), but elevated the abundance of the ribosomal protein L9 (U1). Ribosomal proteins are generally involved in protein synthesis. The impact of azadirachtin on the abundance of ribosomal proteins could suggest that this chemical plays a role in protein biosynthesis.

Several proteins including Bcl-2, NF-κB, P53 and PI3K have been previously suggested to be targets of azadirachtin [37–39]. However, these proteins were not identified in our proteomic analyses. One possible explanation is that the abundance of these regulatory proteins was less affected by azadirachtin, and therefore, were not picked up in our analysis since we did focused on the proteins with most differences. Alternatively, the pH range for our gel strip was pH 4–7, which might not be optimal for separation of these proteins. More studies with different pH ranges should be used to identify other differentially affected proteins after azadirachtin treatments.

## Conclusion

In summary, the present study confirmed that azadirachtin induced apoptosis in Sf9 cells. Azadirachtin-responsive proteins in Sf9 cells were analyzed and 12 differentially expressed protein spots were identified through 2-DE and LTQ-MS/MS analyses. Transcription of the genes encoding Sf-DnaJ1, Sf-PS, Sf-P27BBP/eIF6, Sf-TCTP, Sf-PMSA6 and Sf-AWD was also affected by azadirachtin. In addition, changes in protein levels of Sf-DnaJ1 and Sf-TCTP were confirmed by western blot. Knockdown of the gene encoding Sf-DnaJ1 by RNAi resulted in increased apoptosis induced by azadirachtin, suggesting that Sf-DnaJ1 played an anti-apoptotic role in azadirachtin-induced apoptosis of Sf9 cells. Overall, our results indicated that azadirachtin triggered apoptosis by down-regulating Sf-DnaJ1-encoding gene. Further experiments are needed to obtain more evidence for a more detailed picture on the molecular processes of azadirachtin toxicity on cultured cells and field insects.

## Additional files

Additional file 1: **Table S1.** Details of the primer pairs used for genes cloning, RT-qPCR, RNAi. (DOCX 14 kb)

Additional file 2: **Figure S1.** Z-VAD-FMK inhibited the apoptosis induced by azadirachtin in Sf9 cells. A: Morphological changes induced by different treatments in Sf9 cells. 1, 2, 3 and 4 were represented as normal cells, cells treated with Z-VAD-FMK, cells treated with azadirachtin and cells treated with azadirachtin and Z-VAD-FMK, respectively. B: Caspase-3 like activity induced by different treatments in Sf9 cells. (TIF 3988 kb)

Additional file 3: **Figure S2.** Quantitative analysis of the azadirachtin-responsive proteins in Sf9 cells. The data are expressed as arithmetic mean ± SEM of protein intensity on gels from three independent experiments. Statistical analysis was carried out using the SPSS software and different letters above bars indicate significant differences between different treatments at the same time (*P* < 0.05) by ANOVA followed by DMRT. (TIF 792 kb)

Additional file 4: **Table S2.** The matched peptide sequences of the identified proteins. (DOCX 14 kb)

Additional file 5: **Figure S3.** The coding region sequence, deduced amino acid sequences and phylogenetic analysis of Sf-DnaJ1. A: The coding region sequence and the deduced amino acid sequence of Sf-DnaJ1. B: Phylogenetic analysis of selected DnaJ1. The sequences participate in the phylogenetic tree are: *Linepithema humile* (XP_012222446.1); *Harpegnathos saltator* (XP_011150153.1); *Pogonomyrmex barbatus* (XP_011644113.1); *Apis mellifera* (XP_006566003.1); *Melipona quadrifasciata* (KOX75023.1); *Megachile rotundata* (XP_003699212.1); *Athalia rosae* (XP_012260458.1); *Orussus abietinus* (XP_012274281.1); *Nasonia vitripennis* (XP_008205330.1); *Microplitis demolitor* (XP_008552261.1); *Tribolium castaneum* (XP_971446.1); *Zootermopsis nevadensis* (KDR22500.1); *Plutella xylostella* (XP_011557028.1); *Bombyx mori* (NP_001040292.1); *Amyelois transitella* (XP_013190352.1); *Papilio xuthus* (XP_013165050.1); *Culex quinquefasciatus* (XP_001844792.1); *Aedes aegypti* (ABF18277.1). (PNG 695 kb)

Additional file 6: Figure S4. Silence of DnaJ1 didn’t induce apoptosis in Sf9 cells. Fig A-B. A means control, B shows morphological characteristics of Sf9 cells with ds-DnaJ1 treatment for 24 h. Fig C-D. C means control, D shows the apoptosis of Sf9 cells with ds-DnaJ1 treatment for 24 h, ten thousand cells were counted for each sample. Fig E: Apoptotic rate of Sf9 cells with different treatments. The data represent the mean values± S.E.M of three independent experiments. The apoptotic rate of cells with dsDnaJ1 treatment had no significant difference with normal cells. (TIF 1126 kb)

## Abbreviations

(LTQ)-MS/MS: Linear ion trap quadrupole-mass spectromtry
2-DE: Two-dimensional gel electrophoresis
AIF: Apoptosis inducing factor
DnaJ1: DnaJ homolog subfamily A member1
JNK: c-Jun N-terminal kinase
ROS: Reactive oxygen species
MAPK: Mitogen-activated protein kinase
MALDI-TOF-MS: Matrix-assisted laser desorption/ionization time-of-flight
FBS: Fetal bovine serum
MTT: 3-(4, 5-dimethylthiazol-2-yl)-2,5-diphenyl tetrazolium bromide
PI: Propidium iodide
IAA: Indole-3-acetic acid
IEF: Isoelectric focusing
PVDF: Polyvinylidene difluoride
GAPDH: Glyceraldehyde-3-phosphate dehydrogenase
TCTP: Translationally-controlled tumor protein homolog
Sf-RpL9: Ribosomal protein L9
Sf-AWD: Abnormal wing disc-like protein Sf-RpSA: ribosomal protein SA
Sf-PS: Proteasomezeta subunit
Sf-PMSA6: Proteasome subunit alpha type 6-A
