# Supplementary material for: DnaJ homolog subfamily A member1 (DnaJ1) is a newly discovered anti-apoptotic protein regulated by azadirachtin in Sf9 cells
Source: BMC Genomics. 2018 May 29;19:413. doi: 10.1186/s12864-018-4801-z (PMC5975434; doi:10.1186/s12864-018-4801-z)
Supplement: Supplementary file 1 — Table S1. Details of the primer pairs used for genes cloning, RT-qPCR, RNAi. (DOCX 14 kb) [file 12864_2018_4801_MOESM1_ESM.docx]

Additional file 1: Details of the primer pairs used for genes cloning, RT-qPCR, RNAi.

| **Primers** | **Primers Sequences (5’-3’)** |
| --- | --- |
| **Degenerate Primers**  *Sf-DnaJ1*-F  *Sf-DnaJ1*-R  **RACE Primers**  *Sf-DnaJ1*-5’R  *Sf-DnaJ1*-3’F  **qRT-PCR Primers**  *Sf-DnaJ1*-RT-F  *Sf-DnaJ1*-RT-R  *Sf-PS*-RT-F  *Sf-PS*-RT-R  *Sf-P27BBP/eIF6*-RT-F  *Sf-P27BBP/eIF6*-RT-F  *Sf-TCTP*-RT-F  *Sf-TCTP*-RT-R  *Sf-PMSA6*-RT-F  *Sf-PMSA6*-RT-R  *Sf-AWD*-RT-F  *Sf-AWD*-RT-R  *Sf-GAPDH*-RT-F  *Sf-GAPDH*-RT-R  **RNAi Primers**  ds-DnaJ1-F  ds-DnaJ1-R  ds-GFP-F  ds-GFP-R | CGMATTTAYGAYCAAGGYGG  TGGTTCTTGRTCMCCTTCTC  TGAATCCACCGCCAAAGAACATGTC  CTTCAACTTGGTCAGGTGCC  TTGACAAGGGTATGACTGATGGC  GGAAGCCACACAGTGCCTCAA  GCCATCCAGTTCGGAGACA  AGCCAATAGCCTTAGCATCGT  CAGTCGCAGGCAATGTTTTAG  GCACCAATCGTTGACCACC  GAAGCCGTTGTTTCGGTTTTT  TGCTCTCGTCACCAGTTTGCC  TAAAATGGCACGAGAAAGCAG  CGTTTGGTGAGCGGAAATAG  ATGATTGCCACTTTGCTTTACC  TGTTCCGCCATTACGACTTTT  GTGCCCAGCAGAACATCAT  GGAACACGGAAAGCCATAC  taatacgactcactatagggAGAGCTTGAGCCTGGTGATC  taatacgactcactatagggTTAGCTAGTAGCGCATTGTACTC  taatacgactcactatagggAAGGGCGAGGAGCTGTTCACCG  taatacgactcactatagggCAGCAGGACCATGTGATCGCG |
