# Supplementary figures and images for: DnaJ homolog subfamily A member1 (DnaJ1) is a newly discovered anti-apoptotic protein regulated by azadirachtin in Sf9 cells
Source: BMC Genomics. 2018 May 29;19:413. doi: 10.1186/s12864-018-4801-z (PMC5975434; doi:10.1186/s12864-018-4801-z)

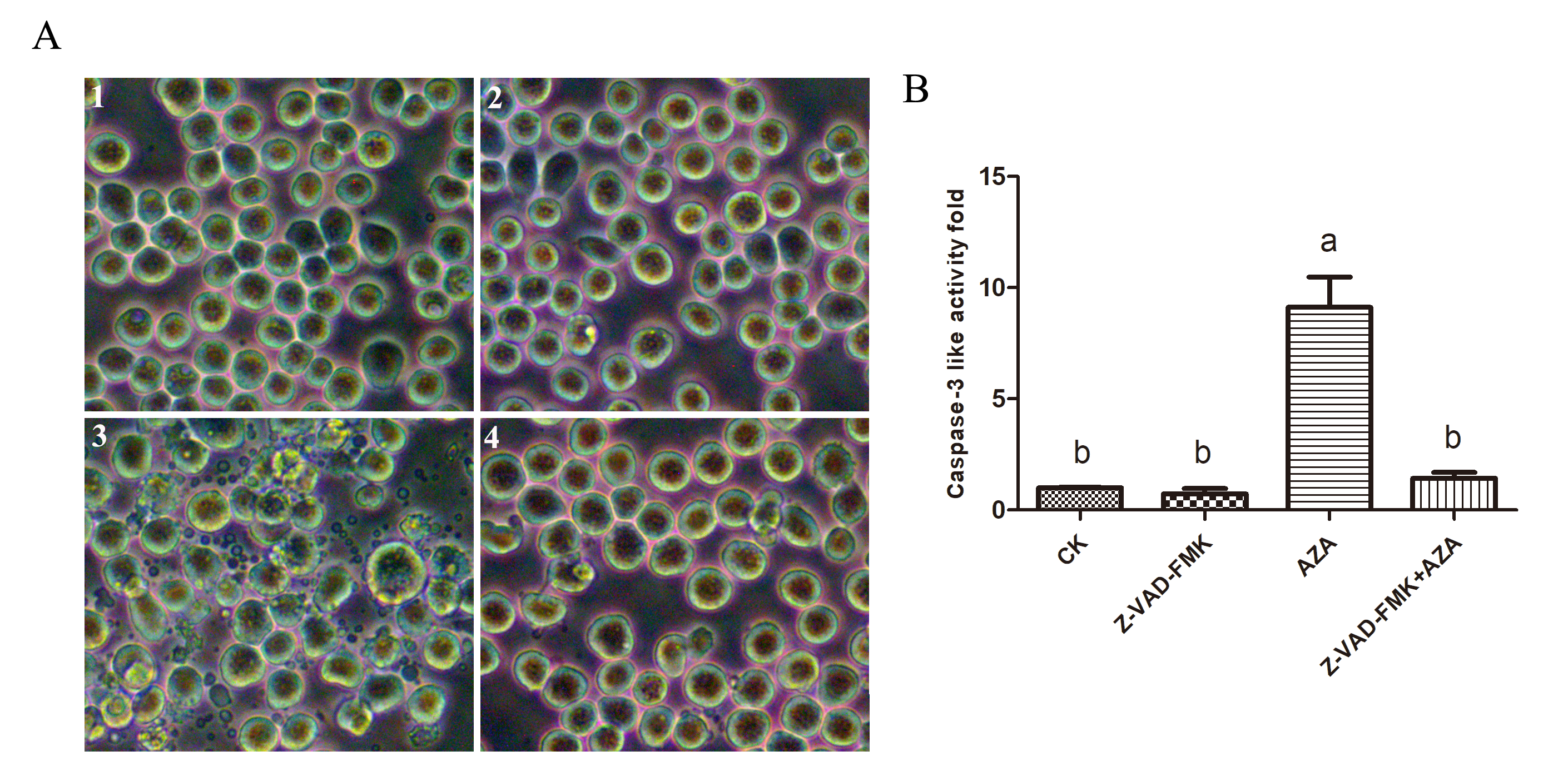

Supplement: Supplementary file 2 — Figure S1. Z-VAD-FMK inhibited the apoptosis induced by azadirachtin in Sf9 cells. A: Morphological changes induced by different treatments in Sf9 cells. 1, 2, 3 and 4 were represented as normal cells, cells treated with Z-VAD-FMK, cells treated with azadirachtin and cells treated with azadirachtin and Z-VAD-FMK, respectively. B: Caspase-3 like activity induced by different treatments in Sf9 cells. (TIF 3988 kb) [file 12864_2018_4801_MOESM2_ESM.tif]

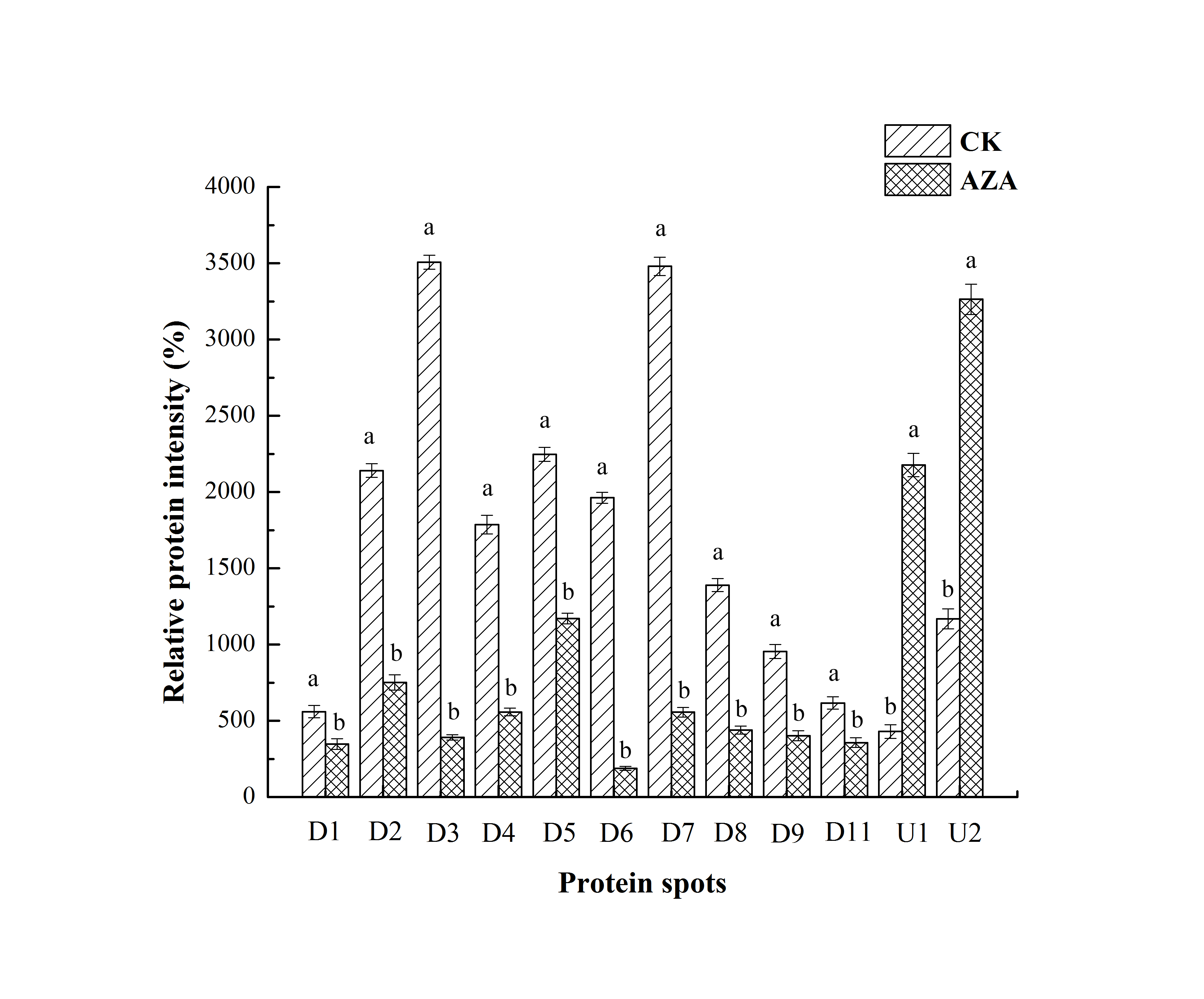

Supplement: Supplementary file 3 — Figure S2. Quantitative analysis of the azadirachtin-responsive proteins in Sf9 cells. The data are expressed as arithmetic mean ± SEM of protein intensity on gels from three independent experiments. Statistical analysis was carried out using the SPSS software and different letters above bars indicate significant differences between different treatments at the same time (P < 0.05) by ANOVA followed by DMRT. (TIF 792 kb) [file 12864_2018_4801_MOESM3_ESM.tif]

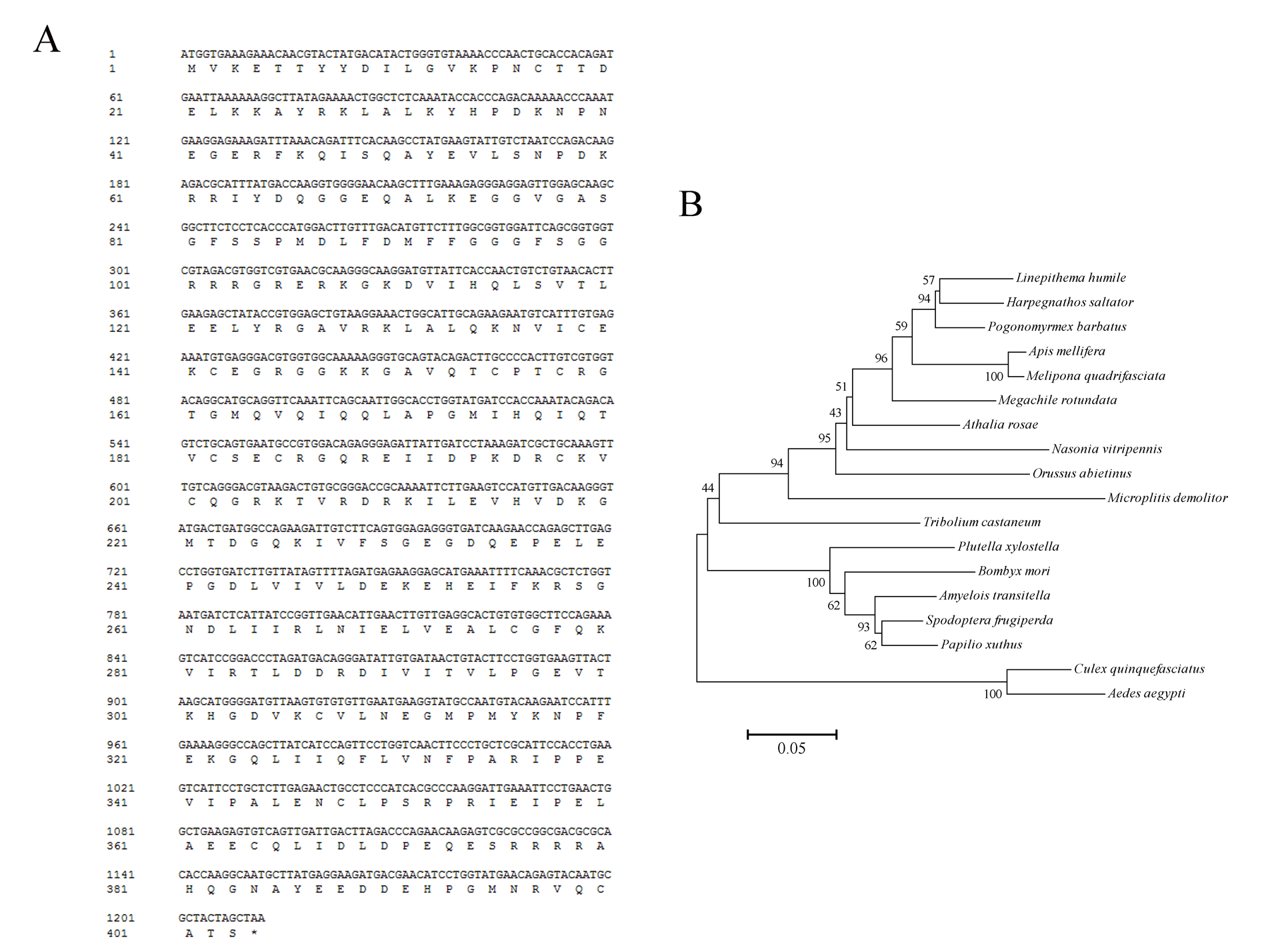

Supplement: Supplementary file 5 — Figure S3. The coding region sequence, deduced amino acid sequences and phylogenetic analysis of Sf-DnaJ1. A: The coding region sequence and the deduced amino acid sequence of Sf-DnaJ1. B: Phylogenetic analysis of selected DnaJ1. The sequences participate in the phylogenetic tree are: Linepithema humile (XP_012222446.1); Harpegnathos saltator (XP_011150153.1); Pogonomyrmex barbatus (XP_011644113.1); Apis mellifera (XP_006566003.1); Melipona quadrifasciata (KOX75023.1); Megachile rotundata (XP_003699212.1); Athalia rosae (XP_012260458.1); Orussus abietinus (XP_012274281.1); Nasonia vitripennis (XP_008205330.1); Microplitis demolitor (XP_008552261.1); Tribolium castaneum (XP_971446.1); Zootermopsis nevadensis (KDR22500.1); Plutella xylostella (XP_011557028.1); Bombyx mori (NP_001040292.1); Amyelois transitella (XP_013190352.1); Papilio xuthus (XP_013165050.1); Culex quinquefasciatus (XP_001844792.1); Aedes aegypti (ABF18277.1). (PNG 695 kb) [file 12864_2018_4801_MOESM5_ESM.png]

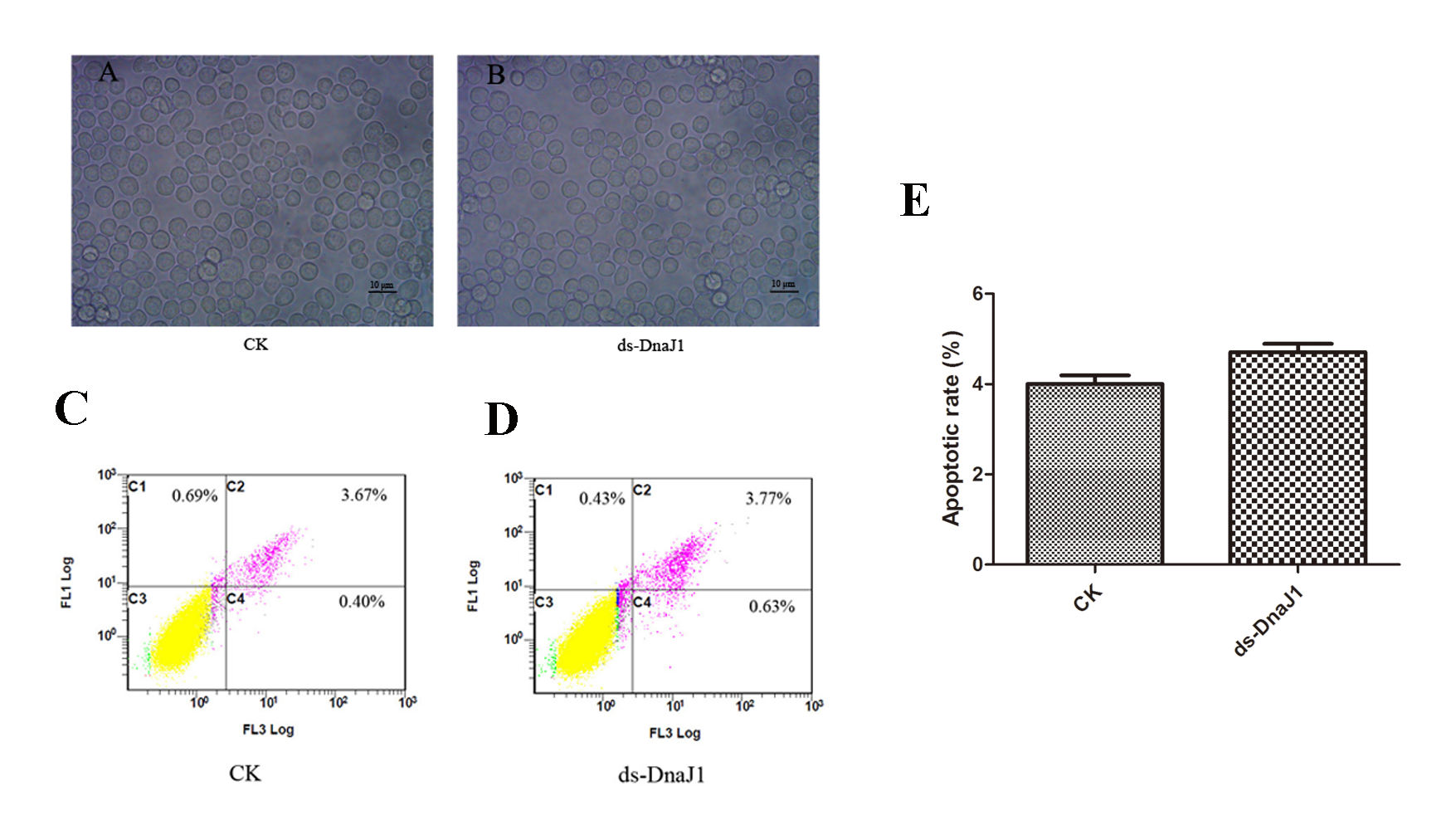

Supplement: Supplementary file 6 — Figure S4. Silence of DnaJ1 didn’t induce apoptosis in Sf9 cells. Fig A-B. A means control, B shows morphological characteristics of Sf9 cells with ds-DnaJ1 treatment for 24 h. Fig C-D. C means control, D shows the apoptosis of Sf9 cells with ds-DnaJ1 treatment for 24 h, ten thousand cells were counted for each sample. Fig E: Apoptotic rate of Sf9 cells with different treatments. The data represent the mean values± S.E.M of three independent experiments. The apoptotic rate of cells with dsDnaJ1 treatment had no significant difference with normal cells. (TIF 1126 kb) [file 12864_2018_4801_MOESM6_ESM.tif]
