# Supplementary material for: DnaJ homolog subfamily A member1 (DnaJ1) is a newly discovered anti-apoptotic protein regulated by azadirachtin in Sf9 cells
Source: BMC Genomics. 2018 May 29;19:413. doi: 10.1186/s12864-018-4801-z (PMC5975434; doi:10.1186/s12864-018-4801-z)
Supplement: Supplementary file 4 — Table S2. The matched peptide sequences of the identified proteins. (DOCX 14 kb) [file 12864_2018_4801_MOESM4_ESM.docx]

Additional file 4: The matched peptide sequences of the identified proteins

| Spot no | DP ^a)^ | Peptides identified |
| --- | --- | --- |
| D1  D2  D3  D4  D5  D6  D7  D8  D9  D11  U1  U2 | 2  3  6  7  7  3  7  4  10  3  6  5 | SGNDLILR DQVEVFK  FAAHTGATPIAGR FAAHTGATPIAGR FVDIAIPCNTK  GYSFTTTAER AGFAGDDAPR CDVDIRK DSYVGDEAQSK DSYVGDEAQSKR EITALAPSTMKI  GYSFTTTAER AGFAGDDAPR DSYVGDEAQSK DLTDYLMK DLTDYLMK EITALAPSTMKI HQGVMVGMGQK  GYSFTTTAER AGFAGDDAPR DSYVGDEAQSK EITALAPSTMKI DLTDYLMK HQGVMVGMGQK DSYVGDEAQSKR  EVDEQMLNIQNK NMMAACDPR TAVCDIPPR  LFQVEYAIEAIK AIGSGSEGAQQSLK GPQLFHMDPSGTFVQYDAK IVEVDR GVNTFSPEGR HIACAVSGLMADSR PFGVAVMFAGIDEK  VQFENNNEVGVFSK LNDAKPTAITSTMR PTAITSTMR ASLIDSMS  LVETYAFGDKK APDQVDIFK DIITGDEMFSDTYK HGLEEEKF KSYTLYLK LEEKAPDQVDIFK LVAKLEEK LVDEVIYEVTGK IYKDIITGDEMFSDTYK LVETYAFGDK  GTDAAVVAAQR AINQGGLTSVALR LYQVEYALK  MAPGVTVVNSPK FLDGLYVSEK KFLDGLYVSEK NFLGEK TVCSHVENMIK MKQIVANQK  NIIHGSDSVESAK GLVGTIIER NIIHGSDSVESAKK PFFPGLVK TFIMVKPDGVQR |
